# Comment on “De Novo Reconstruction of 3D Human Facial Images from DNA Sequence”

**DOI:** 10.1002/advs.202513207

**Published:** 2025-12-07

**Authors:** Jennifer K. Wagner, Nina Claessens, Caitlin M. Maloney, Peter Claes

**Affiliations:** ^1^ School of Engineering Design and Innovation Penn State University 307 EDI Building, 660 White Course Drive University Park PA 16802 USA; ^2^ Department of Electrical Engineering ESAT/PSI KU Leuven Leuven 3000 Vlaams‐Brabant Belgium; ^3^ Medical Imaging Research Center University Hospitals Leuven Leuven 3000 Vlaams‐Brabant Belgium; ^4^ Department of Anthropology Penn State University 237 Susan Welch Liberal Arts Building University Park PA 16802 USA; ^5^ Department of Human Genetics KU Leuven Leuven 3000 Vlaams‐Brabant Belgium

**Keywords:** benchmarking, bioethics, Difface, DNA phenotyping, forensic science, policy, standards

## Abstract

The recent article by Jiao et al., presenting the Difface model for reconstructing faces from deoxyribonucleic acid (DNA) and other artificial intelligence (AI)‐enabled facial genetics research, has transformative potential for biomedical and forensic applications. Yet such research raises serious ethical, legal, and social challenges, as such applications are rights and safety impacting. Methodological transparency, algorithmic explainability, and contextualization of findings are essential to ensure that AI‐enabled facial genetics research, such as Difface, is conducted responsibly and rigorously and interpreted reasonably. Given the lack of standards and benchmarks for DNA‐based face generation, we use Difface as a case study to stress the need for transparent performance metrics and clear disclosure of data flows across AI training, validation, and testing pipelines—enabling non‐experts to assess accuracy meaningfully.

## Graphical abstract

This comment examines AI‐driven DNA‐based facial reconstruction, focusing on the Difface model. While such technologies promise biomedical and forensic applications, they pose significant ethical, legal, and methodological challenges. We emphasize transparency, benchmarking, and rigorous validation to avoid misinterpretation and misuse. Current evidence shows limited accuracy, highlighting the need for cautious deployment and adherence to best practices.

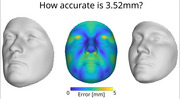

## 1 Main Text

The emergence of AI‐driven DNA‐based facial reconstruction technologies marks a significant milestone in the intersection of genomics, computer vision, and forensic science. Among these, the Difface model introduced by Jiao et al.^[^ ^1^ ^]^ represents a notable advancement, leveraging contrastive learning and diffusion models to generate three‐dimensional (3D) facial images from genetic data. However, the deployment of such technologies in sensitive domains such as law enforcement and healthcare raises pressing ethical, legal, and social concerns.

Given the absence of standardized practices and validated benchmarks in generating facial reconstructions from DNA using AI, there is a serious need to present performance metrics in a way that allows non‐experts and expert users to meaningfully assess performance so that misunderstandings and misapplications can be avoided. Clearly documenting how data is used and flows through AI training and testing pipelines is an essential requirement for AI generally, especially in the sensitive domain of DNA‐based facial phenotyping. Here, using Difface^[^ ^1^ ^]^ as a case study, we offer practical guidance for scientists engaged in facial genetics research to validate and contextualize the performance of the systems developed.

## 2 Difface as a Case Study

**Figure 1:**
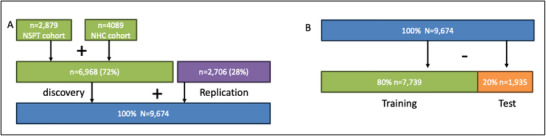
Visualization of cohort description A) and data partitioning B) as given in the methods of Difface. (A) “As a result, 6968 (n = 4089 in the NHC cohort, n = 2879 in the NSPT cohort) and 2706 unrelated individuals with good quality 3D images in the discovery and replication dataset were used for further analysis.” (B) “We utilized a dataset of 9674 samples, each containing 3D facial images, SNP data, age, BMI, and other relevant information. The dataset was randomly split into training and testing sets, with 80% allocated for training and 20% for testing.”.

Difface, developed by Jiao et al.,^[^ ^1^ ^]^ represents a significant step forward in AI‐driven facial phenotyping, offering a multimodal framework that reconstructs 3D facial shapes from DNA using contrastive learning and diffusion models. While the model is trained on a relatively large dataset in facial genetics of Han Chinese individuals (n = 9674, **Figure** **1**A), the genetic input is limited to 7842 common variants identified through a genome‐wide association scan (GWAS) rather than full DNA sequences. The authors leverage contrastive learning, originally used in CLIP (Contrastive Language–Image Pretraining) models trained on hundreds of millions of image‐text pairs,^[^ ^2^ ^]^ to align genetic and facial data in a shared feature space, while the diffusion component^[^ ^3^ ^]^ enhances the generative fidelity of 3D facial reconstructions. Despite the promise of these techniques, the scale of paired genetic and facial data remains limited compared to other domains like vision‐language modelling, raising important questions about how performance is evaluated and communicated. In this context, it becomes essential to scrutinize how the authors present their results and substantiate claims of high accuracy in mapping genetic variation to facial morphology.

To assess the practical utility of Difface, Jiao et al. conduct a series of targeted experiments that span both technical validation and real‐world applicability. These include identity recognition tasks that evaluate how well the model aligns genetic and facial features, as well as quantitative assessments of reconstruction accuracy, both in terms of geometric similarity to ground truth and human recognition performance in lineup scenarios. The authors also examine the model's capacity to predict specific facial traits from DNA, its resilience to reduced SNP input, and its interpretability through explainable AI techniques such as SHAP values,^[^ ^4^ ^]^ which help clarify the contribution of individual genetic variants.

While this broad experimental scope provides a rich understanding of model behavior, it is the core demonstrations, identification, verification, and reconstruction accuracy that are most impactful for practitioners and policymakers seeking to understand the model's real‐world relevance. Unlike reconstruction accuracy, which quantifies how closely a predicted face matches a ground truth shape, identification and verification tasks assess whether the generated face can support recognition of individual identity within a broader population. In verification, the task is to confirm or reject a claimed identity. This is framed as a binary classification problem, where the model must distinguish between genuine identity matches and impostor pairs. Performance is typically evaluated using receiver operating characteristic analysis, with metrics such as the equal error rate (EER) and area under the curve (AUC) providing standardized measures of discriminative ability. Identification, by contrast, involves determining the identity of an unknown individual by comparing a generated face (the probe) against a gallery of known identities. This task answers the question “Who is this person?” and is evaluated using rank‐based metrics, such as rank‐1%, rank‐10%, or rank‐20% accuracy or cumulative match characteristic curves, which reflect how often the correct identity appears among the top k% (k = 1,10, or 20) matches. To be independent of sample size, rank‐based metrics must be expressed as a percentage, instead of absolute rank. These tasks are particularly relevant for assessing real‐world applicability, as they simulate scenarios in which DNA‐derived facial reconstructions might be used for recognition or investigative purposes. As such, they provide a valuable complement to reconstruction accuracy metrics and help clarify the practical boundaries of current model performance. However, these key results warrant clear explanation and benchmarking to ensure that diverse audiences can meaningfully interpret the system's performance.

To support meaningful interpretation of all performance metrics, Jiao et al. follow standard machine learning practice by partitioning their dataset (Figure 1B) into a training (80%, n = 7739) and testing (20%, n = 1935) subset. One of their key experiments evaluates the accuracy of 3D facial reconstruction using different input configurations: single nucleotide polymorphisms (SNPs) alone, demographic variables (age, sex, body mass index), and a combination of both, enabled by the flexible architecture of Difface. The reported average reconstruction errors, 3.52, 7.03, and 2.93 mm, respectively, are measured as Euclidean distances between predicted and actual 3D point clouds. From these results, the authors conclude that SNPs provide a strong foundation for facial reconstruction, while demographic variables alone yield substantially higher errors, underscoring the critical role of genetic information in achieving accurate facial predictions.

While the experimental design is intuitive and the use of millimetre‐scale errors is familiar, the interpretability of these results is limited by missing methodological details and a lack of calibrated (interpretable) errors. First, it is not mentioned explicitly whether the generalized Procrustes analysis used involved size normalization or not. This significantly influences error magnitudes and is essential for contextualizing the reported values. Second, in the absence of standardized benchmarks for DNA‐based facial reconstruction, we propose a simple, yet interpretable reference framework. This benchmark includes three intuitive scenarios: 1) average error between random face pairs (random performance), 2) average error between each face and the population mean face, and 3) average error to the closest face in the dataset (lower error bound for matching different faces). These comparisons help calibrate expectations and define a practical range for interpreting reconstruction accuracies at the millimetre scale. Additionally, we recommend including a simple baseline reconstruction model, e.g., using partial least squares regression (PLSR), using only demographic variables to provide a transparent, interpretable point of comparison for more complex AI systems.

**Figure 2:**
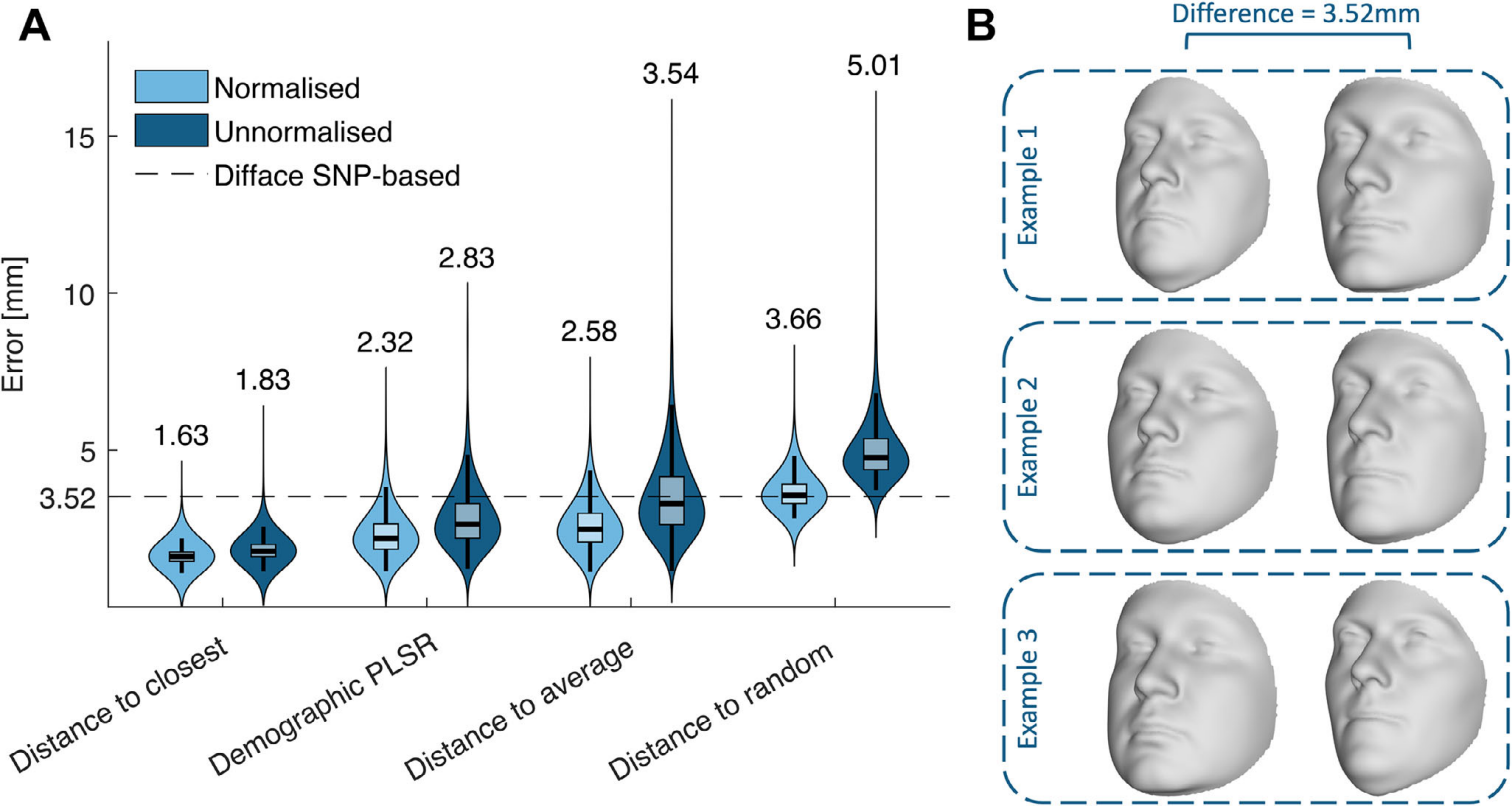
Proposed benchmark for the interpretation of reconstruction accuracies using millimeter‐scale errors. A) Distance to Random, Average, and Closest faces for pairs of faces, and the performance of an interpretable baseline model using demographic predictor variables for facial‐size normalized and unnormalized scenarios. The mean error of each model, in line with the reporting of mean errors in Jiao et al., is displayed above each violin plot, respectively. The mean error of the Difface model, derived from SNP‐based input, is displayed as a striped horizontal line. B) Three synthetically generated face pairs positioned at opposite sides of the population mean, each exhibiting an average Euclidean distance of 3.52 mm.

**Figure** **2** demonstrates the implementation of the proposed benchmarking framework and baseline model using a subset of data previously used in facial GWAS.^[^ ^6^ ^]^ The data consists of 3D facial images from individuals of European descent (n = 4047) above 18 years of age, matched in age range to the dataset used for Difface, and following the same 80–20 data partition for training and testing. Each face was aligned to a standardized 7160‐point facial template using MeshMonk,^[^ ^5^ ^]^ and analyses were conducted on both facial size–normalized and unnormalized data to reflect common preprocessing conditions in facial genetics. As expected, mean reconstruction errors in the test dataset were generally lower for normalized data (Figure 2A). Notably, the mean error between random face pairs, 3.66 mm for normalized and 5.01 mm for unnormalized data, was already relatively low. The test dataset's distance to the average face fell between the random and closest paired scenarios, as anticipated, but was notably very close to the 3.52 mm mean error reported for SNP‐based reconstructions in Difface. For normalized data, this benchmark mean error was even lower than the SNP‐based result in Jiao et al., while the lower mean error bound, defined as the average distance to the most similar face, remained well below all reported Difface mean errors. These findings, although caution in interpretation is warranted due to differences in data composition, suggest that the reconstructions produced by Difface do not yet reach the level of precision required to reliably distinguish between individuals. To visually contextualize this observation, Figure 2B presents three synthetically generated face pairs positioned at opposite sides of the population mean, each exhibiting an average Euclidean distance of 3.52 mm. These examples demonstrate that even faces separated by this seemingly modest error can display substantial morphological differences, underscoring the limitations of current reconstruction precision. Finally, analysis of the simple baseline model employing PLSR with demographic variables revealed a notable reduction in mean reconstruction error, from the level of the dataset's distance to the average face toward the lower bound. This suggests that demographic similarity alone can yield a closer approximation to facial identity, as would be expected from such a straightforward modelling approach. However, this strongly questions the unexpectedly high error (7.03 mm) reported in Jiao et al. for demographic‐only inputs and their conclusion on the critical role of SNPs in facial reconstructions. In addition, it highlights the value and necessity of including transparent, interpretable baselines when evaluating complex AI systems.

**Table** **1** summarizes the performance of our baseline model on both identification and verification tasks, alongside the corresponding results reported for Difface. These experiments move beyond geometric reconstruction accuracy and provide a more applied perspective on the model's potential in biometric contexts, a direction previously explored in related work by Sero et al.^[^ ^7^ ^]^ and Mahdi et al.^[^ ^8^ ^]^ Although evaluated on a different dataset, our baseline model achieves performance levels that are broadly comparable to those reported for Difface, suggesting that demographic information alone can obtain a similar degree of identity matching as Difface. However, these comparisons should be interpreted with caution due to differences in dataset composition. Critically, the absence of a comparable baseline model in the corresponding identification and verification experiments reported by Jiao et al. limits the ability to contextualize Difface's performance relative to simpler, more interpretable approaches.

**Table 1:** Identification and Verification performances of the baseline model using PLSR with demographic variables as input for facial‐size normalized and unnormalized scenarios in comparison to Difface. Verification: EER = equal error rate, AUC = area under the curve. Identification R1% = rank‐1%, R10% = rank‐10% and R20% = rank‐20% Metrics indicated in bold and underlined highlight the best performing model for that metric.

Model	Facial‐size normalized	EER	AUC	R1%	R10%	R20%
Difface	unknown	**27.6**	**80.7**	3.33	23.33	42.53
Demographic PLSR	No	32.83	73.78	**4.20**	**30.53**	**50.56**
	Yes	37.26	67.71	3.96	25.46	42.89

Focusing on the verification performance and more explicitly the EER, which reflects the point at which the false acceptance rate (imposters incorrectly matched) equals the false rejection rate (genuine individuals incorrectly rejected). In this context, an EER of 27.6–32.83%, as observed for both Difface and the baseline model, is considered high. For reference, state‐of‐the‐art biometric systems such as face‐to‐face, fingerprint, or iris recognition routinely achieve EERs below 1%.^[^ ^9^ ^]^ An EER of 25% implies that one in four genuine individuals would be incorrectly rejected, and one in four imposters would be falsely accepted, highlighting the current limitations of DNA‐based facial reconstruction for reliable identity verification. Next, focusing on the identification performance, a rank‐1% of 3.33%‐ 4.20% is considered low. Only 64/34 individuals were identified within the top 1% (top‐n = 19/8) for Difface and our baseline model, respectively, and this out of 1935/809 test cases. Although one should not expect a performance in line with start‐of‐the‐art biometric systems, one should at least illustrate a great improvement against a simple demographic‐based prediction model. These findings underscore the importance of benchmarking against established biometric standards and reinforce the need for cautious interpretation when evaluating the practical readiness of such systems for forensic or security applications. Given these low performance metrics, it is imperative that the discussion and conclusion sections adopt a more measured and critical tone. Such framing is essential to ensure that non‐expert audiences are not misled about the current capabilities of DNA‐based facial reconstruction technologies, particularly in contexts where accuracy and reliability are paramount.

Another experiment introduced by Jiao et al. focuses on evaluating the robustness of Difface under conditions of SNP depletion, a scenario particularly relevant in forensic applications, where DNA samples are often degraded or incomplete, limiting the number of variants that can be reliably genotyped. To simulate this, the authors progressively down‐sampled the full set of 7842 SNPs and assessed the impact on reconstruction accuracy. They report that using 70% of the SNPs (n = 5489) still yields a reconstruction error of 3.71 mm, only slightly higher than the full‐SNP condition. Even when reduced to just 10% of the SNPs, the error increased modestly to 3.96 mm. Based on these results, the authors conclude that Difface maintains a reasonable level of performance under SNP‐limited conditions, suggesting robustness in scenarios where genetic data is incomplete.

However, a critical consideration in interpreting this experiment lies in understanding the structure of the SNP data itself. In GWAS, it is standard practice to group genome‐wide significant SNPs into independent genetic loci, accounting for the correlation between neighboring genetic variants due to linkage disequilibrium. In practice, thousands of significant SNPs often collapse into fewer than 200 independent loci.^[^ ^6^, ^10^, ^11^ ^]^ This raises an important question: to what extent does the full set of 7842 SNPs represent independent sources of information? If most of the predictive power is concentrated in a small number of loci, then randomly retaining 70% of the SNPs is likely to preserve most of the relevant signal. In this case, the observed robustness may reflect redundancy in the data rather than true resilience of the model. Unfortunately, the paper does not provide any details on the genomic structure or independence of the SNPs used, nor does it clarify whether the down‐sampling preserved or disrupted key loci. Without this information, the interpretation of the robustness experiment remains speculative at best and potentially misleading at worst. A more rigorous analysis would require reporting on the distribution of SNPs across loci and assessing whether predictive performance degrades when specific loci are systematically excluded. In other words, the same analysis should be repeated, reporting performance as a function of the percentage of independent loci retained. In the absence of such detail, the robustness claims should be viewed with caution.

To gain further insight into the structure of the SNP data used in the study, we cross‐referenced the cohort descriptions provided in Jiao et al. (Figure 1A) with those reported in Zhang et al., an earlier genome‐wide association study (GWAS) on East Asian facial morphology conducted by some of the same authors.^[^ ^11^ ^]^ Notably, the cohort descriptions are identical across both publications: “As a result, 6968 (n = 4089 in the NHC cohort, n = 2879 in the NSPT cohort) and 2706 unrelated individuals with good quality 3D images in the discovery and replication dataset were used for further analysis.”^[^ ^1^, ^11^ ^]^ This consistency supports the inference that details from the earlier GWAS can inform our understanding of the SNP dataset structure in the current study. Zhang et al. report the identification of 166 independent genetic loci through meta‐analysis, a finding that should be considered when interpreting the SNP deletion experiments. A more robust approach would involve systematically removing entire loci (e.g., 70% then reflects using SNPs lying within 116 out of the 166 independent loci) and observing the resulting degradation in model performance, thereby offering a clearer view of the genetic signal's contribution to facial reconstruction accuracy.

In aligning the cohort descriptions across both publications, a more fundamental methodological concern emerges in the study by Jiao et al. Specifically, there appears to be a clear inconsistency between the reported sample sizes for the discovery and replication phases in Zhang et al. and those used for training and testing in Jiao et al. (See Figure 1). In one plausible scenario, the full set of 7842 SNPs was identified solely within the discovery cohort, which would appropriately inform the training dataset, while the test dataset could correspond to the replication cohort. However, the mismatch in sample sizes suggests that this alignment is unlikely, and that the test dataset does not, in fact, reflect an independent replication cohort. This raises the possibility that SNP selection occurred at the meta‐analysis stage, incorporating data from both discovery and replication cohorts, including individuals later used for testing. Either scenario presents a serious violation of the principle of data independence, which is foundational to machine learning. Both feature selection (i.e., SNP identification by GWAS) and model training (i.e., contrastive learning) must be conducted exclusively on data that is independent of the test set. The available evidence strongly suggests that the test dataset (n = 1935) in Jiao et al. was at least partially involved, if not completely, in the SNP selection process, thereby compromising the integrity of the evaluation. As a result, the reported performance metrics are likely inflated due to overfitting and do not provide a reliable estimate of the model's generalizability. In light of this, it is important for the authors of Jiao et al. to uphold their statement that the data used in their study can be requested, enabling others to independently replicate and validate the reported results.

In conclusion, Difface applies contrastive learning and diffusion models to the task of AI‐driven DNA‐based facial phenotyping. While these techniques represent powerful advances in AI, their application in data‐limited and ethically sensitive domains demands rigorous scrutiny.^17^ Given that most successful AI paradigms are trained on vast, diverse datasets, it is essential to critically evaluate, calibrate, and transparently interpret performance—particularly when the stakes involve identity, privacy, and potential forensic use. Adherence to established best practices in machine learning is not optional but necessary. This begins with the clear separation of training, validation, and test datasets, each serving a distinct role: training data to build the model, validation data to tune hyperparameters, and test data to assess generalizability to unseen cases. Within the context of facial genetics, the discovery cohort in a GWAS may be reasonably aligned with the training set, the replication cohort with the validation set, and a fully independent dataset should be reserved for final testing. Continued vigilance is needed to ensure that forensic science (including but not limited to AI‐driven, DNA‐based facial phenotyping) is valid, reliable, and open to scrutiny.^[^ ^12^, ^13^, ^14^, ^15^, ^16^ ^]^ It is imperative not to approach AI deployment in data‐limited settings prematurely with undue optimism. As our Comment demonstrates, any real‐world deployment of DNA‐based facial phenotyping is *not* yet substantiated by sound science.

## Conflict of Interest

The authors declare no conflict of interest.

## Ethics Approval Statement

The study on the quantification of facial variation and facial genetics was approved by the ethical review board of KU Leuven and University Hospital Leuven (S60568, Leuven, Belgium)

## Data Availability

The facial dataset (n = 4680) was collected previously and included samples from the 3D Facial Norms cohort and studies at the Pennsylvania State University and Indiana University‐Purdue University Indianapolis. Data was collected with informed written consent from all participants. For the 3D Facial Norms dataset, the 3D facial surface models are available through the FaceBase Consortium (FB00000491.01 [https://doi.org/10.25550/VWP]). The participants making up the PSU and IUPUI datasets were previously collected without broad data sharing consent. Given the highly identifiable nature of facial information and unresolved issues regarding risk to participants, a more conservative approach to participant recruitment was opted for. Additional details can be requested from Mark Shriver (mds17@psu.edu) and Susan Walsh (walshsus@iu.edu) for the PSU and IUPUI datasets, respectively.
